# A Simplified Screening Tool for the One-Leg Standing Test to Determine the Severity of Locomotive Syndrome

**DOI:** 10.3390/life13051190

**Published:** 2023-05-16

**Authors:** Takaomi Kobayashi, Tadatsugu Morimoto, Chisato Shimanoe, Rei Ono, Koji Otani, Masaaki Mawatari

**Affiliations:** 1 Department of Orthopaedic Surgery, Faculty of Medicine, Saga University, 5-1-1 Nabeshima, Saga 849-8501, Japan; takaomi_920@yahoo.co.jp (T.K.); mawatam@cc.saga-u.ac.jp (M.M.); 2 Department of Pharmacy, Saga University Hospital, Saga 849-0937, Japan; chisatos@cc.saga-u.ac.jp; 3 Department of Physical Activity Research, National Institute of Health and Nutrition, National Institutes of Biomedical Innovation, Health and Nutrition, 1-23-1, Toyama, Shinjuku-ku, Tokyo 162-8636, Japan; ono@nibiohn.go.jp; 4 Department of Orthopaedic Surgery, Fukushima Medical University School of Medicine, Fukushima 960-1295, Japan

**Keywords:** locomotive syndrome, one-leg standing test, screening, 25-question geriatric locomotive function scale

## Abstract

This study determined the cut-off time for the one-leg standing test (OLST) to simply screen the severity of locomotive syndrome (LS). We conducted this cross-sectional study on 1860 community-dwelling residents (age, 70.5 ± 9.5 years old; males, *n* = 826; females, *n* = 1034) who underwent the OLST and completed the 25-question geriatric locomotive function scale (GLFS-25). Multivariate linear regression and multivariate logistic regression analyses were conducted to assess the relationship between the OLST and the GLFS-25 score and LS after adjusting for age, sex, and body mass index. A receiver operating characteristic (ROC) curve analysis was performed to calculate the optimal cut-off time of the OLST for determining LS severity. The multivariate linear regression and multivariate logistic regression analyses showed that the OLST was significantly associated with the GLFS-25 score and a diagnosis of LS. The optimal cut-off times of the OLST to screen LS-1, LS-2, and LS-3 were 42 s (sensitivity 65.8%, specificity 65.3%), 27 s (sensitivity 72.7%, specificity 72.5%), and 19 s (sensitivity 77.4%, specificity 76.8%), respectively. We developed a simplified screening tool for the OLST to determine LS severity.

## 1. Introduction

**Figure 1:**
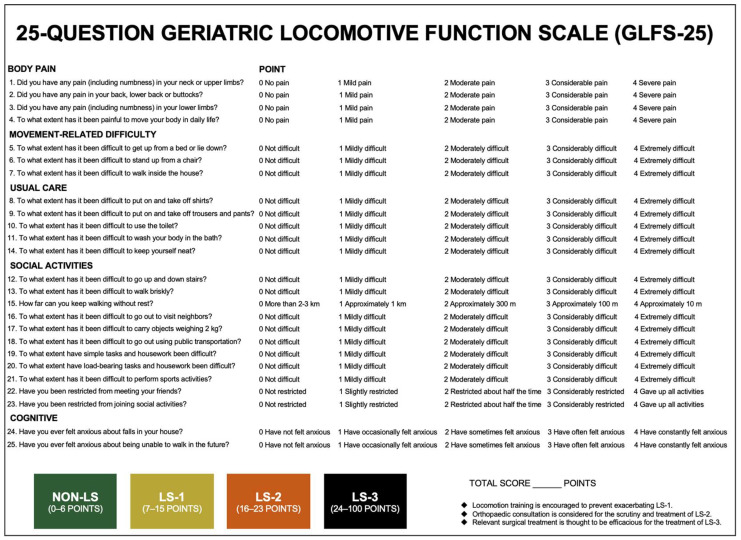
The GLFS-25 questionnaire items. *GLFS*-25, 25-question geriatric locomotive function scale; *LS*, locomotive syndrome.

With the aging of society, the Japanese Orthopaedic Association (JOA) has spread the concept of ‘locomotive syndrome’ (LS) and developed a diagnostic tool called the 25-question geriatric locomotive function scale (GLFS-25) [1,2,3]. The GLFS-25 includes 25 questionnaire items graded on a 5-point scale, for a total possible score of 0–100 (Figure 1); total scores of 0–6 points, 7–15 points, 16–23 points, and 24–100 points are defined as non-LS, LS-1, LS-2, and LS-3, respectively [2,3]. Locomotion training is encouraged to prevent exacerbating LS-1, orthopedic consultation is considered for the scrutiny and treatment of LS-2, and relevant surgical treatment is thought to be efficacious for the treatment of LS-3 [3].

However, despite the precision of the GLFS-25 and its management, its complexity remains a major limitation, especially for elderly individuals, and the response rate reportedly ranges from 50% to 70% [4,5]. Accordingly, the relationship between the GLFS-25 and various physical performance tests (e.g., grip strength, maximum stride, timed up-and-go, one-leg standing test [OLST], and gait speed) has been investigated [6,7,8,9,10,11,12,13].

Among them, the OLST is one of the easiest physical performance tests to perform [6,7,8,9,10,11,12]. Furthermore, the OLST does not require special testing equipment and it can be performed easily by anyone. However, while some investigators have assessed the best cut-off times of the OLST for diagnosing LS-2 [6,7,8], the results have varied. Furthermore, the cut-off time of the OLST for screening LS severity (i.e., LS-1, LS-2, and LS-3) in community-dwelling residents remains entirely unexplored. These problems may hinder the clinical application of the GLFS-25. The present study explored the optimal cut-off times of the OLST for simply screening LS severity in community-dwelling residents.

## 2. Materials and Methods

### 2.1. Study Design

The study was approved by the institutional ethics committee. The description of this paper was followed according to the standards for reporting diagnostic accuracy (STARD) [14]. We conducted a cross-sectional study on Japanese volunteers (≥40 years old) who were living in their own houses and able to walk independently and attended a ‘basic health checkup’ in Minami-Aizu Town and Tadami Town in Fukushima Prefecture, Japan, in 2017. The exclusion criteria were as follows: (1) individuals who did not provide their written informed consent to be interviewed; (2) individuals who did not complete all questions of the GLFS-25; (3) individuals who had comorbidities (i.e., cerebrovascular diseases, cardiovascular diseases, pulmonary diseases, and/or renal diseases [15]); and (4) individuals without evaluable data.

**Figure 2:**
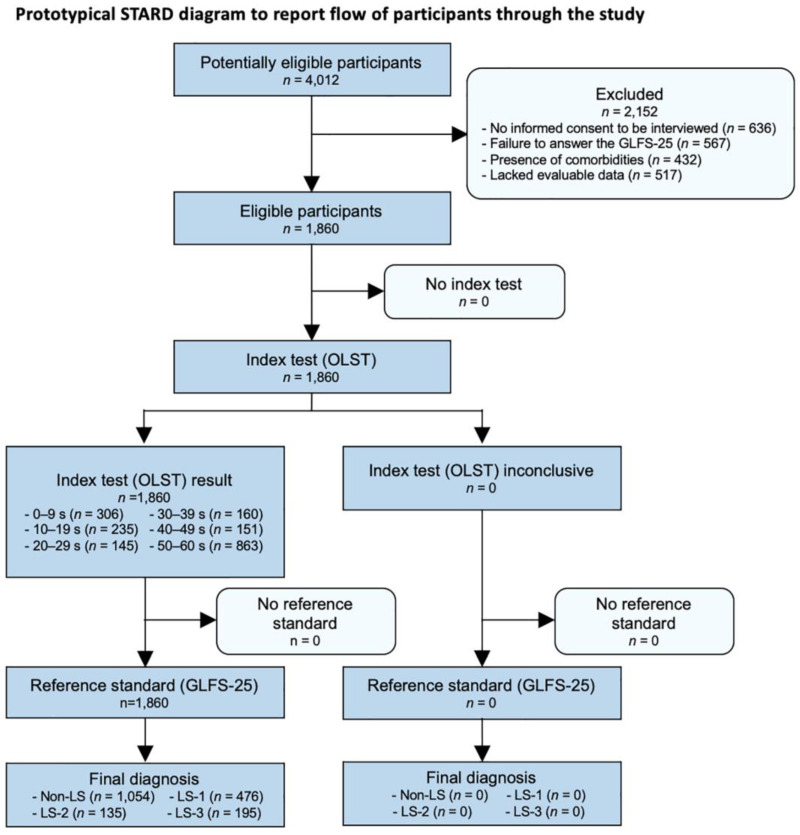
Prototypical STARD diagram reporting the flow of participants through the study. *STARD*, standards for reporting diagnostic accuracy; *GLFS-25*, 25-question geriatric locomotive function scale; *LS*, locomotive syndrome; *the OLST*, one-leg standing test.

Among 4012 potentially eligible participants, individuals who did not provide their written informed consent to be interviewed (*n* = 636), did not complete all questions of the GLFS-25 (*n* = 567), had comorbidities (*n* = 432), and lacked evaluable data (*n* = 517) were excluded (Figure 2). Ultimately, 1860 (male, *n* = 826; female, *n* = 1034; mean age, 70.5 ± 9.5 years old) were considered eligible for this study.

### 2.2. GLFS-25

The GLFS-25 includes 25 questionnaire items, all of which feature a 5-point scale (Figure 1): no impairment—0 points, mild impairment—1 point, moderate impairment—2 points, considerable impairment—3 points, and severe impairment—4 points. The total possible score ranges from 0 to 100. The domain scores include body pain (items 1–4), movement-related difficulty (items 5–7), usual care (items 8–11 and 14), social activities (items 12, 13, and 15–23), and cognition (items 24 and 25). A GLFS-25 total score of 0–6 points, 7–15 points, 16–23 points, and 24–100 points were categorized into non-LS, LS-1, LS-2, and LS-3, respectively [2,3].

### 2.3. The OLST

The OLST was conducted with the patients’ eyes open (and their hands on their hips) once for each leg. The test was continuously performed in the same time slot. The OLST was recorded with a stopwatch to measure the duration from when the subject raised his/her leg until their leg was set back down on the floor (up to 60 s [s]). We recorded the average time of the two measurements., i.e., average OLST = (right OLST + left OLST)/2.

### 2.4. Statistical Analyses

We used the JMP^®^ pro 16 software program (SAS Institute, Cary, NC, USA) for all analyses in this study. We set the level of significance (*p*-value) at 0.05. For assessments of the association between the GLFS-25 scores and the OLST, we conducted a simple regression analysis to calculate the crude regression coefficient (β) and a multivariate linear regression analysis with the ordinary least squares method to calculate the adjusted β controlled for age (years, continuous), sex (0: male, 1: female), and body mass index (kg/m^2^, continuous). We used the GLFS-25 scores (points, continuous) as a dependent variable and the OLST (s, continuous) as an independent variable. For determinations of the association between the diagnosis of LS and the OLST findings, we conducted a univariate logistic regression analysis to calculate crude odds ratios (ORs) and a multivariate logistic regression analysis to calculate adjusted ORs that were controlled for age (years, continuous), sex (0: male, 1: female), and body mass index (kg/m^2^, continuous). We used the LS (LS-1 or more [0: absent, 1: present], LS-2 or more [0: absent, 1: present], or LS-3 or more [0: absent, 1: present]) as a dependent variable and the OLST (s, continuous) as an independent variable. We conducted a receiver operating characteristic (ROC) curve analysis to calculate the optimal cut-off time for the OLST for identifying individuals with LS-1 or more, LS-2 or more, and LS-3 or more, with a preference for slightly higher sensitivity, as the tool is primarily intended for screening purposes. As in previous reports [7,8], a gender analysis was also performed. We assessed the discriminative ability of the model according to the area under the ROC curve (AUC). The AUC values of 0.50–0.59, 0.60–0.69, 0.70–0.79, 0.80–0.89, and 0.90–1.00 were classified as failure, poor, fair, good, and excellent, respectively [16].

## 3. Results

### 3.1. Participants

Of the 1860 participants, 1054 (56.7%), 476 (25.6%), 135 (7.3%), and 195 (10.5%) were diagnosed with non-LS, LS-1, LS-2, and LS-3, respectively (Table 1). The median OLST was 45 s (interquartile range, 15–60 s).

### 3.2. Regression Analyses

The univariate and multivariate linear regression analyses showed that the OLST had significant associations with the GLFS-25 domain scores (i.e., movement-related difficulty, usual care, social activity, and cognition) and the total score (Table 2). The univariate and multivariate logistic regression analyses showed that the OLST had significant associations with the diagnoses of LS-1 or more, LS-2 or more, and LS-3 or more (Table 3).

### 3.3. ROC Analyses

**Figure 3:**
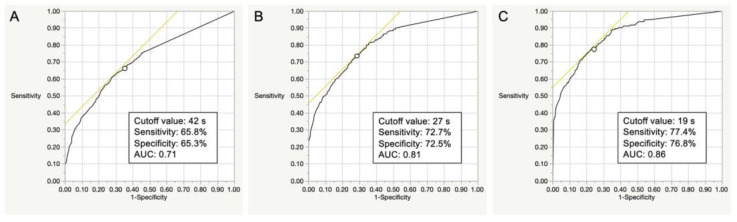
ROC analyses of the OLST to determine the optimal cut-off values for identifying LS-1 (**A**), LS-2 (**B**), and LS-3 (**C**) in the overall participants. *ROC*, receiver operating characteristic; *AUC*, area under the ROC curve; *OLST*, one-leg standing test; *LS*, locomotive syndrome.

In all participants, the discriminative ability of the OLST model was considered fair to good (AUC 0.71–0.86). The optimal cut-off times of the OLST to screen LS-1 or more, LS-2 or more, and LS-3 or more were 42 s (sensitivity 65.8%, specificity 65.3%), 27 s (sensitivity 72.7%, specificity 72.5%), and 19 s (sensitivity 77.4%, specificity 76.8%), respectively (Figure 3).

**Figure 4:**
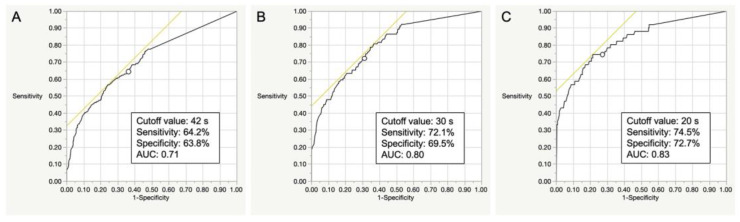
ROC analyses of the OLST to determine the optimal cut-off values for identifying LS-1 (**A**), LS-2 (**B**), and LS-3 (**C**) in the male participants. *ROC*, receiver operating characteristic; *AUC*, area under the ROC curve; *OLST*, one-leg standing test; *LS*, locomotive syndrome.

In the male participants, the discriminative ability of the OLST model was considered fair to good (AUC 0.71–0.83). The optimal cut-off times of the OLST to screen LS-1 or more, LS-2 or more, and LS-3 or more were 42 s (sensitivity 64.2%, specificity 63.8%), 30 s (sensitivity 72.1%, specificity 69.5%), and 20 s (sensitivity 74.5%, specificity 72.7%), respectively (Figure 4).

**Figure 5:**
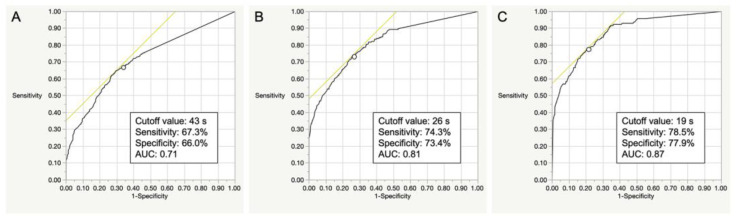
ROC analyses of the OLST to determine the optimal cut-off values for identifying LS-1 (**A**), LS-2 (**B**), and LS-3 (**C**) in the female participants. *ROC*, receiver operating characteristic; *AUC*, area under the ROC curve; *OLST*, one-leg standing test; *LS*, locomotive syndrome.

In the female participants, the discriminative ability of the OLST model was considered fair to good (AUC 0.71–0.87). The optimal cut-off times of the OLST to screen LS-1 or more, LS-2 or more, and LS-3 or more were 43 s (sensitivity 67.3%, specificity 66.0%), 26 s (sensitivity 74.3%, specificity 73.4%), and 19 s (sensitivity 78.5%, specificity 77.9%), respectively (Figure 5).

## 4. Discussion

To our knowledge, this is the first study to calculate the optimal cut-off time of the OLST for screening LS severity. Our main findings were as follows: (1) the OLST had significant associations with the GLFS-25 domain scores and the total score and the diagnoses of LS and (2) the optimal cut-off times of the OLST to screen LS-1 or more, LS-2 or more, and LS-3 or more were approximately 40 s, 30 s, and 20 s, respectively.

We found that the OLST had significant associations with the GLFS-25 domain scores and the total score and the diagnoses of LS. Similar to our findings, past studies observed a significant relationship between the OLST and the GLFS-25 total score [6,7,8,9,10,11,12]. Indeed, the OLST is thought to indicate both static (holding our body in a specific position) and dynamic balance function (maintaining balance while moving our body and walking–e.g., risk of falls [17,18,19,20]), a decrease in which leads to a decline in movement-related difficulty [21,22], usual care [21,22], social activity [21,22], and cognition [23]. Therefore, the OLST has significant associations with the GLFS-25 domain scores of movement-related difficulty (items 5–7), usual care (items 8–11 and 14), social activity (items 12, 13, and 15–23), and cognition (items 24 and 25), resulting in a clear association between the GLFS-25 total score and a related diagnosis of LS.

We found that the optimal cut-off times of the OLST to screen LS-1 or more, LS-2 or more, and LS-3 or more in community-dwelling residents (age, 40–96 years old) were approximately 40 s, 30 s, and 20 s, respectively. However, previous studies detected varied results (Table 4). Seichi et al. [6] found that the optimal cut-off times of the OLST to detect LS-2 or more were 19 s (sensitivity 69%, specificity 65%, AUC 0.73) in those 65–70 years old, 10 s (sensitivity 70%, specificity 71%, AUC 0.76) in those 71–75 years old, and 6 s (sensitivity 70%, specificity 67%, AUC 0.76) in those 75–96 years old. Muramoto et al. [7] found that the optimal cut-off times of the OLST to detect LS-2 or more were 21 s (sensitivity 71%, specificity 73%, AUC 0.75) in males and 15 s (sensitivity 69%, specificity 74%, AUC 0.78) in females. Nakamura et al. [8] found that the optimal cut-off time of the OLST to detect LS-2 or more was 15 s (sensitivity 57.1%, specificity 93.8%, AUC 0.74) in females.

These inconsistencies may be multifactorial, with possible responsible factors including differences in the subjects, procedures, and statistical analyses. To be more detailed, the subjects were not unified, including the participants who attended a basic health checkup (age, 40–96) in this study, outpatients of clinics and hospitals (age, 65–96 years old) [6], the participants who attended a basic health checkup (age, 60–88 years old) [7], and the female participants who attended a basic health checkup (age, 34–84 years old) [8]. The measurements of the OLST were also not unified, including average time (once on each leg) [6], average time (twice on each leg) [7], and maximum time (once on each leg) [8]. Approaches to selecting a cut-off point on an ROC curve were also not unified, including a cut-off point where the sum of sensitivity and specificity was maximal [6], where sensitivity and specificity had similar values [7], and where false negatives and false positives had similar values [8]. Given the above, we recommend using our approach as a screening tool for community-dwelling residents.

**Figure A1:**
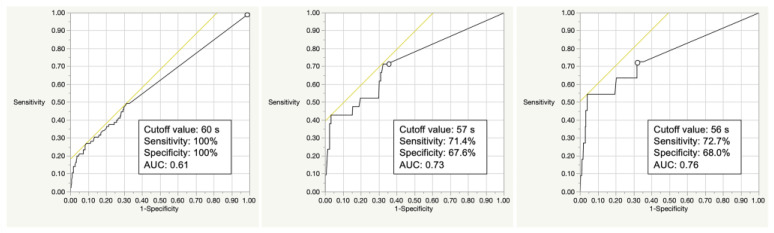
ROC analyses of the OLST to determine the optimal cut-off values for identifying LS-1, LS-2, and LS-3 in males 40–70 years old. *ROC*, receiver operating characteristic; *OLST*, one-leg standing test; *LS*, locomotive syndrome; *AUC*, area under the ROC curve.

**Figure A2:**
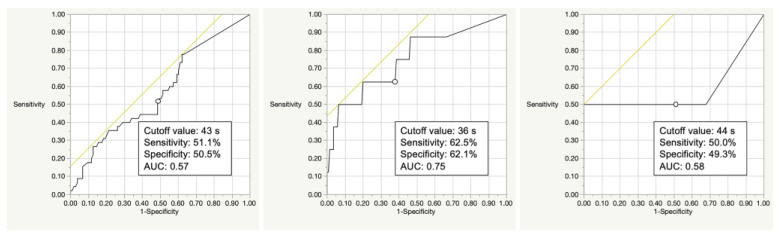
ROC analyses of the OLST to determine the optimal cut-off values for identifying LS-1, LS-2, and LS-3 in males 71–75 years old. *ROC*, receiver operating characteristic; *OLST*, one-leg standing test; *LS*, locomotive syndrome; *AUC*, area under the ROC curve.

**Figure A3:**
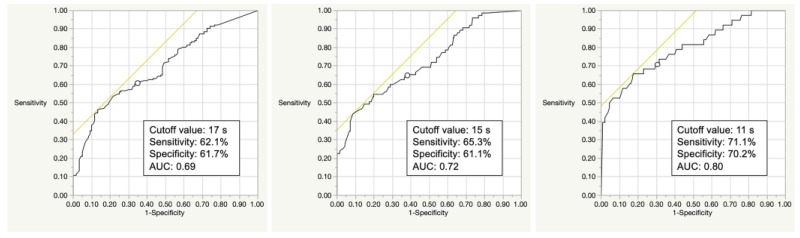
ROC analyses of the OLST to determine the optimal cut-off values for identifying LS-1, LS-2, and LS-3 in males ≥ 76 years old. *ROC*, receiver operating characteristic; *OLST*, one-leg standing test; *LS*, locomotive syndrome; *AUC*, area under the ROC curve.

**Figure A4:**
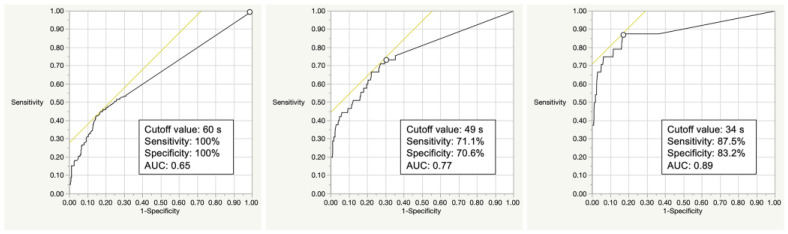
ROC analyses of the OLST to determine the optimal cut-off values for identifying LS-1, LS-2, and LS-3 in females 40–70 years old. *ROC*, receiver operating characteristic; *OLST*, one-leg standing test; *LS*, locomotive syndrome; *AUC*, area under the ROC curve.

**Figure A5:**
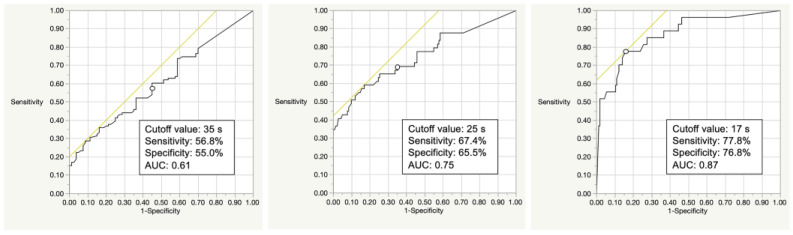
ROC analyses of the OLST to determine the optimal cut-off values for identifying LS-1, LS-2, and LS-3 in females 71–75 years old. *ROC*, receiver operating characteristic; *OLST*, one-leg standing test; *LS*, locomotive syndrome; *AUC*, area under the ROC curve.

**Figure A6:**
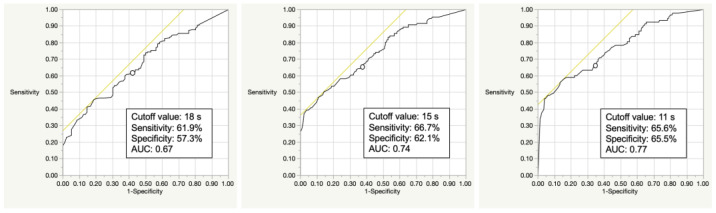
ROC analyses of the OLST to determine the optimal cut-off values for identifying LS-1, LS-2, and LS-3 in females ≥ 76 years old. *ROC*, receiver operating characteristic; *OLST*, one-leg standing test; *LS*, locomotive syndrome; *AUC*, area under the ROC curve.

To provide the Supplementary Information, we measured the cut-off time of the OLST according to age and sex (Appendix A, Figure A1, Figure A2, Figure A3, Figure A4, Figure A5 and Figure A6) in the same classification as Seichi et al. [6]. The optimal cut-off time differs among the age and sex groups. To be more precise, older age was associated with a lower cut-off time, and the cut-off time for the male participants was higher than that for the female participants in those ≥71 years old. Nevertheless, these results may be too complex to use in the clinical field. When we use the simplified screening tool (cut-off time of the OLST for identifying LS-1 or more, 40 s; LS-2 or more, 30 s; and LS-3 or more, 20 s) regardless of age and sex, LS severity may be underestimated in those 40–70 years old and overestimated in those ≥76 years old. Importantly, the rates of LS-1 or more increased with age (Table 1), with them being 261/916 (27.2%) in those 40–70 years old, 156/339 (46.0%) in those 71–75 years old, and 389/605 (64.3%) in those ≥76 years old. From the viewpoint of screening, the simplified screening tool may be clinically useful regardless of age or sex. However, 6.0% (60/1005) of the participants with an OLST of >40 s were diagnosed with LS-2 or more (Appendix A, Table A1), so we should bear in mind that further assessments with official diagnostic tools (i.e., the GLFS-25, the two-step test, and the stand-up test [3]) are necessary after using this simplified screening tool.

Several limitations associated with this study warrant being mentioned. First, we calculated only the average times of the OLST on both legs. It seems difficult to accurately evaluate body balancing by an evaluation at the maximum or minimum time. For instance, if one leg is normal and the OLST is 60 s while the other leg suffers from knee osteoarthritis and the OLST is 20 s, the evaluation at the maximum value overestimates to 60 s or at the minimum value, it underestimates to 20 s. Both legs are required for standing and walking, so the average time should be evaluated. Second, sample bias may have affected our results. All eligible participants were able to walk by themselves and answer the GLFS-25. Therefore, their physical performance tests may have been better than those of average elderly individuals. Third, the test–retest reliability and interrater reliability were not assessed in this study. However, in previous studies, these values were reported to be acceptable [21,24,25]. Fourth, 60 s was adopted as the maximum time for the OLST in this study, which led to a non-normal distribution of the parameter. However, this measurement method has been traditionally used in previous studies [6,26,27], and it is clinically difficult to test for more than 60 s on both legs from a simplicity standpoint. Finally, and most importantly, the present study developed a simplified screening tool without validation. Additional validation studies with a different population are therefore necessary.

## 5. Conclusions

We developed a simplified screening tool for community-dwelling residents (≥40 years old); the optimal cut-off times of the OLST to screen LS-1 or more, LS-2 or more, and LS-3 or more were approximately 40 s, 30 s, and 20 s, respectively. It is necessary to clinically apply this simple screening tool for the early detection, prevention, and treatment of LS.

## Figures and Tables

**Table 1:** Clinical characteristics of the eligible participants in this study.

Demographic	Overall (*n* = 1860)	Male (*n* = 826)	Female (*n* = 1034)
Age, years	70.5 ± 9.5	70.9 ± 9.8	70.1 ± 9.1
40–70 years old			
Non-LS, *n* (%)	655 (35.2)	307 (37.2)	348 (33.7)
LS-1, *n* (%)	195 (10.5)	64 (7.7)	131 (12.7)
LS-2, *n* (%)	31 (1.7)	10 (1.2)	21 (2.0)
LS-3, *n* (%)	35 (1.9)	11 (1.3)	24 (2.3)
71–75 years old			
Non-LS, *n* (%)	183 (9.8)	103 (12.5)	80 (7.7)
LS-1, *n* (%)	99 (5.3)	37 (4.5)	62 (6.0)
LS-2, *n* (%)	28 (1.5)	6 (0.7)	22 (2.1)
LS-3, *n* (%)	29 (1.6)	2 (0.2)	27 (2.6)
≥76 years old			
Non-LS, *n* (%)	216 (11.6)	120 (14.5)	96 (9.3)
LS-1, *n* (%)	182 (9.8)	91 (11.0)	91 (8.8)
LS-2, *n* (%)	76 (4.1)	37 (4.5)	39 (3.8)
LS-3, *n* (%)	131 (7.0)	38 (4.6)	93 (9.0)
Body mass index, kg/m^2^	23.6 ± 3.3	23.8 ± 2.9	23.5 ± 3.6
GLFS-25 total score, points	5 (2–12)	4 (1–9)	6 (3–14)
GLFS-25 domain score, points			
Body pain	2 (1–4)	2 (0–4)	3 (1–5)
Movement-related difficulty	0 (0–0)	0 (0–0)	0 (0–1)
Usual care	0 (0–0)	0 (0–0)	0 (0–0)
Social activities	2 (0–6)	1 (0–5)	3 (1–7)
Cognition	0 (0–1)	0 (0–1)	0 (0–2)
OLST, s	45 (15–60)	47 (17–60)	43 (15–60)

*GLFS-25*, 25-question geriatric locomotive function scale; *LS*, locomotive syndrome; *OLST*, one-leg standing test. The values represent the mean ± standard deviation or median (interquartile range). The classification of age and sex is the same as that used by Seichi et al. [6].

**Table 2:** Univariate and multivariate linear regression analyses on the relationship between the GLFS-25 and the OLST.

Dependent Variable	Independent Variable	Crude *β* (95% CI)	*p*-Value	Adjusted *β* * (95% CI)	*p*-Value
GLFS-25 total score, points	OLST, s	−0.24 (−0.26 to −0.22)	<0.001	−0.19 (−0.21 to −0.16)	<0.001
GLFS-25 domain score, points					
Body pain	OLST, s	−0.03 (−0.04 to −0.02)	<0.001	−0.02 (−0.03 to −0.02)	<0.001
Movement-related difficulty	OLST, s	−0.02 (−0.03 to −0.02)	<0.001	−0.02 (−0.02 to −0.01)	<0.001
Usual care	OLST, s	−0.03 (−0.03 to −0.02)	<0.001	−0.02 (−0.03 to −0.02)	<0.001
Social activities	OLST, s	−0.13 (−0.15 to −0.12)	<0.001	−0.11 (−0.12 to −0.10)	<0.001
Cognition	OLST, s	−0.02 (−0.02 to −0.01)	<0.001	−0.02 (−0.02 to −0.01)	<0.001

*GLFS-25*, 25-question geriatric locomotive function scale; *OLST*, one-leg standing test; *β*, regression coefficient; *CI*, confidence interval. * Adjusted for age (years, continuous), sex (0: male, 1: female), and body mass index (kg/m^2^, continuous).

**Table 3:** Univariate and multivariate logistic regression analyses on the relationship between the LS and the OLST.

Dependent Variable	Independent Variable	Crude OR (95% CI)	*p*-Value	Adjusted OR * (95% CI)	*p*-Value
LS-1 or more	OLST, s	0.96 (0.96 to 0.97)	<0.001	0.98 (0.97 to 0.98)	<0.001
LS-2 or more	OLST, s	0.95 (0.94 to 0.95)	<0.001	0.96 (0.95 to 0.97)	<0.001
LS-3 or more	OLST, s	0.93 (0.92 to 0.94)	<0.001	0.94 (0.93 to 0.95)	<0.001

*LS*, locomotive syndrome; *OLST*, one-leg standing test; *OR*, odds ratio; *CI*, confidence interval. *Adjusted for age (years, continuous), sex (0: male, 1: female), and body mass index (kg/m^2^, continuous).

**Table 4:** Summary of previous reports regarding cut-off values of the OLST for LS-2 or more.

Study	Subject (Number of Subjects)	Body Mass Index (kg/m^2^)	Cut-off Time (s)	Sensitivity (%)	Specificity (%)	AUC
Seichi et al., 2014 [6]	Overall (*n* = 880)	NA	9	71	72	0.79
	65–70 years (*n* = 142)	NA	19	69	65	0.73
	71–75 years (*n* = 234)	NA	10	70	71	0.76
	≥76 years (*n* = 504)	NA	6	70	67	0.76
Muramoto et al., 2013 [7]	Male (*n* = 167)	24.0 ± 2.9	21	71	73	0.75
	Female (*n* = 239)	23.5 ± 3.4	15	69	74	0.78
Nakamura et al., 2015 [8]	Female (*n* = 126)	23.3 ± 3.0	15	57	94	0.74
Present study	Overall (*n* = 1860)	23.6 ± 3.3	27	73	73	0.81
	Male (*n* = 826)	23.8 ± 2.9	30	72	70	0.80
	Female (*n* = 1034)	23.5 ± 3.6	26	74	73	0.81

*OLST*, one-leg standing test; *LS*, locomotive syndrome; *AUC*, area under the receiver operating characteristic curve; *NA*, not available.

## Data Availability

The datasets used during the current study are not publicly available because of patient confidentiality but are available from the corresponding author on reasonable request.

## Appendix A

**Table A1:** The relationship between the simple screening test of the OLST and the distribution of LS diagnoses.

	OLST			
	0–20 s (*n* = 556)	21–30 s (*n* = 145)	31–40 s (*n* = 154)	41–60 s (*n* = 1005)
Non-LS (*n* = 1054)	183	65	89	717
LS-1 (*n* = 476)	156	50	42	228
LS-2 (*n* = 135)	65	15	14	41
LS-3 (*n* = 195)	152	15	9	19

*OLST*, one-leg standing test; *LS*, locomotive syndrome.
